# Of Viroids and Prions

**DOI:** 10.3390/v10120663

**Published:** 2018-11-23

**Authors:** Theodor O. Diener

**Affiliations:** Department of Molecular Genetics, University of Maryland, College Park, MD 20472, USA; tod26@juno.com

**Keywords:** viroids, prions, RNA, subviral pathogens, HIV

## Abstract

In 2017, Hadidi et al. edited a voluminous monograph entitled “Viroids and Satellites”, in which each known viroid and viroid-like satellite species was described in detail from many perspectives by more than 100 experts from 24 countries. In its 700+ pages, the book is a much needed detailed and reliable compendium of a subject, which, undoubtedly, is still little known by many potential readers. Because most users of the book may be expected to be practical plant pathologists, it appears essential that the book contain, in addition to the detailed viroid and satellite descriptions, one chapter, in which the basic molecular biology of viroids and satellites is described.

## 1. Viroids

In 2017, Hadidi et al. [1] edited a voluminous monograph entitled “Viroids and Satellites”, in which each known viroid and viroid-like satellite species was described in detail from many perspectives by more than 100 experts from 24 countries. In its 700+ pages, the book is a much needed detailed and reliable compendium of a subject, which, undoubtedly, is still little known by many potential readers.

Because most users of the book may be expected to be practical plant pathologists, it appears essential that the book contain, in addition to the detailed viroid and satellite descriptions, one chapter, in which the basic molecular biology of viroids and satellites is described.

While, traditionally, this is the task of an introductory chapter called “Introduction”, the chapter so named in this book is mostly composed of the invited author’s “personal reflections and musings of past and present that may be questioned as any review or book chapter might”. Unfortunately, no other chapter fills in for the missing information. Also, strangely for an introduction, the author does not name the scientist who is universally recognized as the discoverer of the viroid.

In its preface, the editors of the same book incorrectly state that Theodor O. Diener of the Department of Agriculture, Beltsville, Maryland discovered the infectious nature of the potato spindle tuber disease—presumably by showing transmissibility of the disease from diseased to healthy plants by rubbing sap from diseased plants onto healthy plants, and demonstrating that the inoculated plants developed the characteristic disease syndrome—thus proving the infectious nature of the disease, a result already achieved by Schultz and Folsom in the 1920s [2]. The editors, however, correctly state that Diener discovered that its pathogenic agent (Potato spindle tuber viroid, PSTVd) is “a free RNA…much smaller than any viral genome”, which he named viroid, but not in 1972, as stated, but in 1971 [3]. The editors, however, correctly state that, in 1972, Joseph S. Semancik of the University of California, Riverside showed that the causing agent of the citrus exocortis disease is also a viroid [4].

It was, however, not the less than one-year difference between Diener’s and Semancik’s discoveries alone that established Diener universally as the discoverer of the viroid, but the fact that Diener (together with William Raymer) [5] in 1967 already obtained convincing evidence that PSTVd is a protein-free RNA 50–80 times smaller than the smallest viral genomes. However, I realized at that time already that my conclusions were subject to alternative, if less likely, interpretations, such as, for example, that the apparently unbelievably small size of the PSTVd genome might be an illusion, brought on by the presence in our seemingly monodisperse peaks or gradient fractions of several RNAs of similar size which, after inoculation, could assemble head-to-toe into a viral genome of conventional size.

It was this uncertainty that prevented me for almost four years from publishing a definite version of my discovery. Rapidly encroaching competition—some achieved with my innocently provided help—forced me to announce [3] what was later declared by the American Phytopathological Society to be one of the six most important pathogen discoveries of the millennium [6].

By then, I also knew that PSTVd was not the only extant viroid, but that there existed a still unknown empire of such subviral pathogens. Accordingly, I decided that these novel entities deserved a name. After much cogitation, I thought of viroid and wondered whether it was used previously. After two days (and half-nights) without hits, I almost gave up. However, while scanning the American Naturalist, I came across prominent geneticist Edgar Altenburg’s viroid theory [7]. Although there is hardly a recognizable conceptual connection between his viroid and PSTVd, I wrote to Dr. Altenburg, but was disappointed and saddened when my letter was returned with the message “addressee deceased”. Because Altenburg’s viroid theory is not mentioned in any modern biology textbooks I scanned, to prevent confusion, I decided not to cite it in my publications either.

Dr. Semancik, on the other hand, who also came across Altenburg’s viroid theory, thought it to have sufficient scientific significance to be mentioned in his “Introduction”. He considered the reintroduction of the term viroid—which Edgar Altenburg coined in 1946 to describe “possible…symbionts akin to viruses…in animal cells”, an invaluable stimulus promoting research on the small pathogenic RNAs identified in the early 1970s. While Dr. Semancik could not have known that I coined the term viroid independently of Altenburg’s, I am certain, now that he knows, he will give me credit for having introduced the term viroid independently in 1971 and for the thereby accruing “invaluable stimulus”.

At that time (1975), I was contacted by a representative of the Alexander von Humboldt Foundation, who informed me that I won their Alexander von Humboldt Award, but only if I would agree to share it equally with Dr. Joseph Semancik, because Dr. Semancik’s work—which shortly after my initial report provided additional independent evidence of the existence and pathogenicity of viroids by studying a different disease and on a woody host—may have been instrumental in having viroids more easily accepted by a scientific community not yet prepared for this unexpected discovery. Therefore, I was delighted to include Dr. Semancik as an equal in the von Humboldt Award and immediately accepted it.

## 2. Prions

Starting in the early 1960, I followed the efforts of several medical scientists to isolate and biochemically characterize the infectious agent of a well-known infectious sheep disease, scrapie. While some of these efforts led to interesting results, none had yet achieved their ultimate goal.

After I identified the infectious agent of the Potato spindle tuber disease as the first representative of the first order of subviral agents now named viroids—representing the third major enlargement of the biosphere in history to smaller entities, after the discovery of the microorganisms by A. van Leeuwenhoek in 1675 and of the viruses by D. I. Ivanovski in 1892—I was surprised to learn that apparently no scrapie investigators knew of viroids, or considered viroids as a possible model of the scrapie agent. I decided to write a theoretical piece, in which I would suggest that the scrapie agent might be a small, unencapsidated RNA akin to a viroid—which, in light of my results with an infectious plant disease, was not then an impossible proposition [8]. This time, the reaction was swift and led to a visit by Dr. Stanley Prusiner, then of the National Institutes of Health (NIH), who was a newcomer among the scientists trying to determine the biochemical nature of the scrapie agent.

Dr. Prusiner complimented me on my scientific work and particularly for naming PSTVd a viroid, which he thought greatly accelerated the acceptance of subviral agents. He told me that his lab was at an advanced stage in the purification of the scrapie agent and that my experience with naming viroids encouraged him to name his partially purified preparation prion, which he told me was to mean “proteinaceous infectious particle”.

Whereas, aside from a few raised eyebrows, the term viroid was readily accepted by the plant virological community, Prusiner’s naming of his highly purified scrapie preparations prion caused a veritable firestorm (as he vividly describes in his autobiography [9]), partly because he was not naming a precisely known structure, and partly because the concept of an infectious particle without nucleic acid was a priori an unacceptable concept to many investigators.

Dr. Prusiner introduced me to the intricacies of the scrapie infectious agent and particularly to the controversies surrounding the results of efforts to determine its biochemical nature, which indicated that the agent did not contain nucleic acid—an unacceptable result to many investigators, who were only willing to accept the conventional nucleic-acid-containing virus model to explain the infectivity of scrapie.

It occurred to me that maybe my viroid work could clarify the then confused scrapie situation, and proposed a collaborative effort between the two labs. Dr. Prusiner immediately accepted and we started designing experiments in which we compared the viroid with the scrapie agent.

Essentially, we mixed known amounts of purified PSTVd with Prusiner’s prion preparation and determined whether the mixtures were infectious for both PSTVd (in tomato seedlings in my lab in Beltsville) and scrapie (with a recently developed hamster test in Prusiner’s lab at NIH). To obtain meaningful results, all experiments were severalfold repeated. We obtained the plant results in about three weeks, but had to be patient for more than a year for obtaining the hamster results [10]. These were unequivocal; whenever the viroid was inactivated by a procedure that modified RNA, the infectivity of the prion was preserved. Each time the prion was inactivated by a procedure that modified protein, the infectivity of the viroid was maintained. As expected, the viroid provided an important control. Not only were prions not viruses, they were also not viroids. Our collaborative work, entitled “Viroids and Prions”, was published in the Proceedings of the National Academy of Sciences in September 1982—the first paper in which the term prion appeared in the title [10].

While our results unequivocally demonstrated that prions were devoid of any recognizable nucleic acid, “they did not put an end to the debate about whether or not scrapie was caused by a virus, perhaps one composed of a small nucleic acid protected by a thick protein coat” [10]. Dr. Prusiner spent many more years searching for the mythical nucleic acid of the prion [10]. Why? As he stated, “if my conclusion that prions were devoid of nucleic acid was to be overturned, I wanted to be the one to do it” [10].

His revolutionary claim that the scrapie agent, despite its infectious nature, does not contain nucleic acid stood the test of time. By then, I had sufficiently interacted with Stan to know that, below his sometimes rather aggressive and overly ambitious façade, lay a true scientist, who, like few others, has a free mind and is never bound by preconceived ideas or concepts.

I wish to end with a little amusing incident. When Stan invited his Washington/Baltimore friends to a sumptuous celebration of his now being a Nobel laureate, after which we all stood in line to retrieve our coats, I felt a slight tap on my shoulder and turned around. There was a prominent virologist, famous for his work in identifying the aids virus, telling me: “too bad, Ted, they don’t go into humans”.
